# A new perspective on NO pathway in sepsis and ADMA lowering as a potential therapeutic approach

**DOI:** 10.1186/s13054-022-04075-0

**Published:** 2022-08-12

**Authors:** Jaipal Singh, Young Lee, John A. Kellum

**Affiliations:** 1 Indiana Center for Biomedical Innovation, Indiana University School of Medicine, 1800 N. Capitol Ave, Noyes Building, E504C, Indianapolis, IN 46202 USA; 2 Center for Critical Care Nephrology, Department of Critical Care Medicine, University of Pittsburg, Pittsburg, PA USA; 3 Vasculonics Inc., Indianapolis, IN USA

## Abstract

The nitric oxide pathway plays a critical role in vascular homeostasis. Increased levels of systemic nitric oxide (NO) are observed in preclinical models of sepsis and endotoxemia. This has led to the postulation that vasodilation by inducible nitric oxide synthase (iNOS) generated NO may be a mechanism of hypotension in sepsis. However, contrary to the expected pharmacological action of a nitric oxide synthase (NOS) inhibitor, clinical studies with L-NAME produced adverse cardiac and pulmonary events, and higher mortality in sepsis patients. Thus, the potential adverse effects of NO in human sepsis and shock have not been fully established. In recent years, the emerging new understanding of the NO pathway has shown that an endogenously produced inhibitor of NOS, asymmetric dimethylarginine (ADMA), a host response to infection, may play an important role in the pathophysiology of sepsis as well as organ damage during ischemia–reperfusion. ADMA induces microvascular dysfunction, proinflammatory and prothrombotic state in endothelium, release of inflammatory cytokines, oxidative stress and mitochondrial dysfunction. High levels of ADMA exist in sepsis patients, which may produce adverse effects like those observed with L-NAME. Several studies have demonstrated the association of plasma ADMA levels with mortality in sepsis patients. Preclinical studies in sepsis and ischemia–reperfusion animal models have shown that lowering of ADMA reduced organ damage and improved survival. The clinical finding with L-NAME and the preclinical research on ADMA “bed to bench” suggest that ADMA lowering could be a potential therapeutic approach to attenuate progressive organ damage and mortality in sepsis. Testing of this approach is now feasible by using the pharmacological molecules that specifically lower ADMA.

## Background

The host immune response in sepsis and septic shock is accompanied by major hemodynamic, microcirculatory and metabolic changes that may consequently lead to impaired tissue oxygenation, multi-organ damage and death. Reduced blood pressure is a hallmark of sepsis even in the absence of shock [1]. Systemic increase in nitric oxide (NO) in response to inflammation contributes to vasodilation, whereas reduced NO induces microvascular dysfunction. The former has led to the hypothesis that the inhibition of NO generation would restore blood pressure and improve outcome in sepsis. This hypothesis led to the clinical testing of nitric oxide synthase (NOS) inhibitor L-NAME as a potential treatment for sepsis. The initial preclinical and clinical studies showed that L-NAME produced hemodynamic benefit by increasing blood pressure. However, despite the improved blood pressure, treatment with L-NAME was associated with reduced cardiac output, increased pulmonary artery pressure, end-organ damage and mortality [2, 3]. The adverse effects of reduced NO bioavailability have been further confirmed in subsequent studies in which NO was reduced by treatment with NO scavenger pyridoxylated hemoglobin polyoxyethylene (PHP). In a phase-3 clinical trial, treatment with PHP showed evidence of increased mortality in patients with a SOFA score of > 13. It was thought that regional dysregulation NO synthesis in the microcirculation could have been exacerbated in the PHP-treated patients [4]. These clinical [2–4] and other preclinical studies [5] clearly showed that the inhibition of NO synthesis is detrimental to patients with sepsis. The detrimental effect of L-NAME may be attributed to the disruption of the critical role of NO in microvascular homeostasis, which can lead to microvascular constriction, leukocyte adhesion, red blood cells deformation and adhesion, platelet activation and the formation of microthrombosis. The impaired microvascular blood flow and O_2_ delivery result in accelerated organ damage.

Since the initial studies with L-NAME, significant advances have been made on the understanding the molecular mechanisms of the NO pathway at the level of systems biology. In particular, the endogenously produced inhibitor of nitric oxide synthase, asymmetric dimethylarginine (ADMA) and its pathological activities have emerged as an important arm of the NO-pathway biology [6–8]. Importantly, in addition to inducing endothelial dysfunction, ADMA can produce deleterious effects on cellular and mitochondrial function of the target organs. High ADMA levels are found in sepsis and other forms of critically ill patients. In preclinical studies, increased ADMA as a result of the deletion of the ADMA-metabolizing enzyme dimethylarginine dimethylaminohydrolase (DDAH) gene dramatically reduced survival in polymicrobial sepsis model [9], whereas reduction of ADMA by increased expression of DDAH-2 resulted in organ protection and improved survival [10]. Our recent studies have demonstrated that ADMA levels in the blood can be reduced by pharmacological treatment with recombinant DDAH molecule, which resulted in reduced tissue injury in the animal models of renal and cardiac ischemia [11, 12]. Thus, a new perspective on the role of NO pathway in sepsis and ADMA lowering as a potential target of therapy has emerged.

## Main text

### NO changes in human sepsis

The initial data that led to the clinical evaluation of L-NAME for sepsis treatment were largely derived from studies in rodent models of sepsis, which showed generation of high levels of NO by iNOS induction [5]. However, similar results on the magnitude of iNOS induction have not been observed in large animal species models as well as human studies [5]. Instead, studies using arginine flux measurements have presented evidence that systemic production of NO in sepsis and shock patients either does not change or may actually decrease [13–15]. The observed increase in plasma nitrate, a measure commonly used to reflect NO production, is likely due the impairment of renal function and altered the volume of distribution of nitrate/nitrite in sepsis [15]. The evidence of reduced NO bioavailability in human sepsis is also suggested by the reduced NO-dependent reactive hyperemia as measured by peripheral arterial tonometry [16]. The already reduced NO in the microvascular compartment and its further reduction by treatment with L-NAME may have contributed to the observed deleterious effects in sepsis patients. Thus, the observations of high levels of NO generation and its potential deleterious effects in rodent models are not directly translatable to human disease. Instead, reduced NO bioavailability due to exogenous L-NAME or endogenous ADMA may exacerbate pathological responses in sepsis.

### Pathological effects of ADMA

**Fig. 1:**
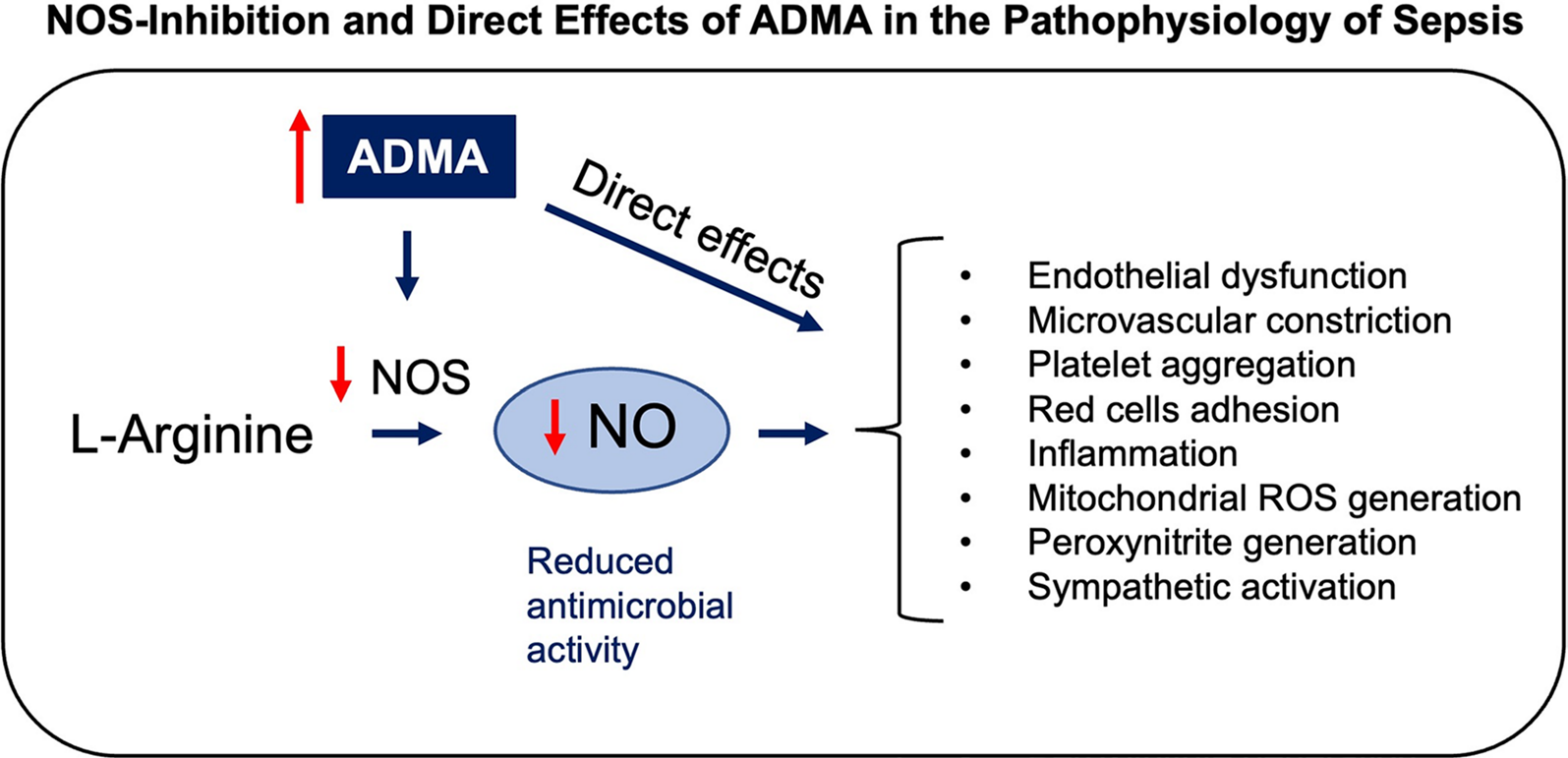
The activities of ADMA which can induce pathological state in the microcirculation leading to reduced perfusion and O_2_ delivery to the organs. These actions of ADMA are mediated through decrease in NO or independent of NOS inhibition. ROS: reactive oxygen species

High levels of ADMA accumulate under the conditions of ischemia, inflammation and oxidative stress, which commonly occur in sepsis. Under these conditions, ADMA is released as a result of protein degradation [6]. At the same time, the expression and the activity of the ADMA metabolizing enzyme DDAH are reduced, leading to the observed increase in plasma ADMA. Since active transport of ADMA occurs through the cation transporter in the cell membrane and mitochondria [17, 18], the intracellular levels of ADMA are more than 10 times that seen in plasma. Thus, high levels of ADMA in the blood may be an indicator of much higher levels within the tissues. The high ADMA levels could produce a variety of pathological responses including endothelial dysfunction [19], platelet aggregation [20], immune cell adhesion to endothelium [21], endothelial cytokine release, vascular leak [10], red cell deformity [22], generation of reactive oxygen species (ROS) [23], peroxynitrite production [24] and sympathetic activation [25] as summarized in Fig. 1.

### ADMA levels and sepsis mortality

The pathological activities of ADMA are particularly relevant to sepsis. Multiple studies have shown that high concentrations of ADMA are present in the blood of sepsis patients [26–34]. More importantly, ADMA levels in patients with sepsis correlate with disease severity (higher in septic shock) and mortality [26, 30, 33]. ADMA is a strong predictor of 30-day mortality in sepsis [28]. Several studies have proposed ADMA as a potential biomarker of sepsis-associated mortality [28–30, 33, 35, 36]. High levels of ADMA are also associated with the hemodynamic and cardiovascular perturbations, which occur in sepsis including pulmonary hypertension [37], increased incidence of arrhythmia [38], microcirculatory disturbances, myocardial dysfunction and impaired regional tissue perfusion and end organ damage [39, 40]. In addition, other studies have shown occurrence of high levels of ADMA in critically ill patients and its association with mortality [41–43].

Consistent with the above clinical studies, high levels of ADMA are produced in preclinical models of endotoxemia [10]. Deletion of DDAH-2 gene in an animal model of sepsis increased ADMA and produced excess mortality as compared to the wild-type animals [9]. The deleterious activities of ADMA in the vasculature and in the target end-organs suggest that pathological levels of ADMA may be a risk factor for organ failure.

Endothelial dysfunction is a prominent feature of sepsis pathophysiology [44]. The prothrombotic and proinflammatory state of endothelium with the expression of endothelial adhesion molecules and loss of vascular tight junction leads to enhanced coagulopathy and inflammatory cell infiltration into the tissues. Loss of endothelial permeability barrier enhances microvascular leakage, tissue edema, organ damage and shock. Besides these effects of ADMA on endothelial dysfunction, our recent studies have shown that ADMA induces inflammatory cytokines IL-6 and IL-17 release from endothelial cells and impairs mitochondrial energy metabolism [11]. High levels of IL-6 are produced in sepsis patients, which remains high for more than a week. IL-6 levels correlate with plasma ADMA, severity of sepsis and mortality [45]. IL-6 also affects capillary permeability and coagulation by increasing plasminogen activator inhibitor-1 (PAI-1) [46, 47]. Further, inflammasome NLRP3 activation induces caspase-1-dependent mitochondria damage and opening of the mitochondrial permeability transition pores (mPTPs) [48]. Thus, the sustained high levels of ADMA may play important role in the pathogenesis of sepsis progression.

The role of ADMA in microvascular dysfunction and organ damage has been illustrated in preclinical models of ischemia–reperfusion such as in acute kidney and myocardial injury [49–53] or type 2 diabetes [54]. As indicated in Fig. 1, high ADMA levels in the setting of sepsis may produce multiple adverse effects on microvasculature leading to hypoperfusion. These effects of ADMA may be mediated by impairment of NO in the microvasculature as well as its direct effects on the bioenergetics of the cells of the end-organs such as cardiomyocytes [11, 55].

### Benefits of increased NO bioavailability in sepsis

Several lines of evidence suggest that improved NO bioavailability may provide therapeutic benefit to microvasculature. In animal models, administration or arginine, inorganic nitrites, NO donors and NO gas has been shown to improve microvascular function and reduce damage in response to ischemia–reperfusion injury to the heart, lung and kidney. Circulating arginine is reduced in response to infections [13, 56] as a result of reduced de novo synthesis and increased catabolism by arginase [57]. Arginine administration has shown benefit in preclinical [58, 59] and some clinical [60–62] studies. Direct administration of NO via an inhaled route has recently been tested in patients with sepsis [63]. NO administration increased circulating nitrite levels consistent with the hypothesis that pulmonary delivery of NO could lead to increased systemic availability. However, the potential efficacy of arginine or NO supplementation during sepsis and in critically ill patients remains to be confirmed using optimum drug-able approaches. More recently, sustained delivery of exogenous NO using NO-releasing nanoparticles was shown to improve microvascular flow and capillary transit, reduce inflammation, and significantly improve 72-h survival of mice after LPS-induced endotoxemia [64]. Although these studies provide encouraging preclinical and early clinical evidence for a beneficial response to improving NO bioavailability, there remain significant challenges to the development of these molecules as therapeutics [65]. The challenges include drug stability (e.g., inhaled NO), drug delivery and dose, and their potential adverse effects under oxidative stress. Further understanding of the optimal dose, route and intervention schedule in sepsis patients may help clarify their potential efficacy. It is also important to note that in the presence of high levels of ADMA, administration of arginine could lead to excessive production of organ damaging ROS by endothelial NO synthase (eNOS) uncoupling and peroxynitrite formation. Also, ADMA has been shown to directly reduce phosphorylation of eNOS and exacerbate vascular damage in eNOS-deficient mice [66]. It is plausible that arginine or NO-donor treatments may be more beneficial when ADMA levels are reduced.

### ADMA lowering as potential therapeutics

**Fig. 2:**
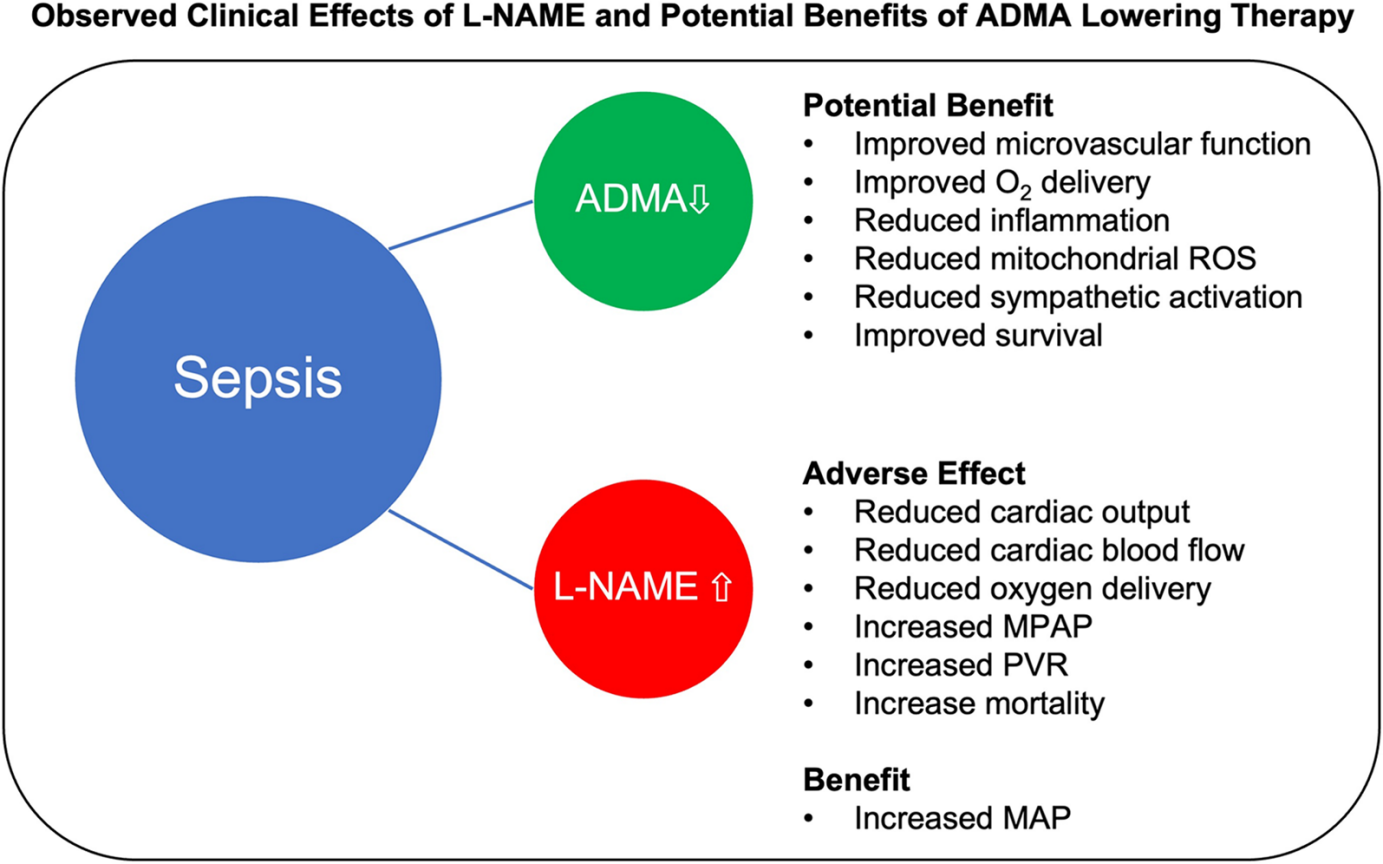
The potential benefits of ADMA lowering based on the preclinical studies and the adverse effects of L-NAME observed in clinical trials. ROS: reactive oxygen species, MPAP: Mean pulmonary arterial blood pressure, PVR: Pulmonary vascular resistance, MPA: Mean arterial pressure

Alternative to arginine or NO donors, the specific lowering of ADMA appears a highly promising and safe approach for improving NO bioavailability as well as mitigating the other deleterious effects of ADMA. The adverse effects from a short time exposure to L-NAME clearly suggest that persistent high levels of endogenous ADMA could similarly contribute to the pathological response and mortality in sepsis. These adverse effects of L-NAME and the potential benefits of lowering ADMA, as documented in a variety of preclinical studies, are listed in Fig. 2. We hypothesize that reduction in pathological levels of ADMA in sepsis patients may improve microvascular function, oxygen delivery, metabolic function, and reduce organ damage. Our hypothesis is based on the human data on L-NAME and translating it back to the development of new therapies based on reducing the endogenously produced ADMA.

Since ADMA levels are predominantly regulated by DDAH [6, 7], this enzyme is an important target for reducing pathological ADMA. It has been shown that the deletion of DDAH-1 gene in animals produced higher levels of ADMA and vascular dysfunction [19]. Alternatively, overexpression of DDAH-1 in transgenic animals or by adenoviral gene transfer reduced ADMA, improved vascular responses and reduced disease severity [50, 51, 67]. Induction of lung injury in mice by LPS was associated with increased ADMA and reduced DDAH activity. In this model, overexpression of DDAH reduced ADMA, lung vascular leak, restored lung injury and improved outcome in LPS-induced organ damage [10]. Similarly, in animal models of ischemia–reperfusion injury, DDAH gene delivery significantly protected heart, kidney, liver and lung [50, 51]. More recently, our studies have used pharmacological treatment with DDAH protein, which effectively and specifically reduced ADMA and offered significant protection to kidney [12] and heart [11] from ischemia–reperfusion injury. Thus, lowering of ADMA could produce therapeutic benefit by preserving microvascular function and improving organ perfusion. This is also consistent with the observation that ADMA reduces the efficacy of resuscitation. In a study using the porcine model of hemorrhagic septic shock, it was found that the efficacy of resuscitation was correlated with the arginine/ADMA values. Pigs with low arginine/ADMA exhibited reduced microcirculation and poor response to resuscitation. The lower arginine/ADMA showed worse microvascular blood flow in the kidney, small and large intestine, and needed greater resuscitation volumes [68].

It is important to note that some studies have indicated that increasing ADMA may be a therapeutic strategy for sepsis. In a preclinical study, an inhibitor of DDAH was shown to improve indices of renal and hepatic function, preserve microvascular flow and improve survival without effect on immune cell function [69]. It is, however, not clear how increasing the levels of ADMA, a non-selective inhibitor of NOS, could produce a different outcome than that observed with L-NAME. Since pharmacological compounds can produce off-target effects, it is not clear whether the beneficial effects observed in this study were specific to DDAH inhibition.

At the present time, the therapeutic modalities to lower pathological ADMA are not available. We recently showed that a biotherapeutic approach by administering DDAH protein can effectively and specifically lower ADMA. ADMA lowering with DDAH produced significant protection of kidney and heart in response to ischemia–reperfusion injury [11, 12]. Our studies have also shown that treatment with DDAH reduced inflammatory cytokines and mitochondrial ROS generation [11]. Based on the pharmacological proof of efficacy, we have also developed an extracorporeal device that can effectively lower ADMA in pigs when used in conjunction with a plasmapheresis system [70]. The therapeutic extracorporeal device (TED) option offers a highly safe and effective approach to specifically lower ADMA**.** Using TED, ADMA can be lowered without drug exposure to the patient. The treatment can be tailored to individual patients by calibrating the duration of plasmapheresis and the plasma flow rate. Furthermore, unlike a drug molecule which can last for a long time in the body, ADMA lowering by TED can be terminated as needed. These recently developed pharmacological molecule and extracorporeal device represent important tools to test the potential benefits of ADMA lowering.

### Safety of therapeutic improvement of NO in sepsis

Our therapeutic strategy is not to produce excessive increase of NO, rather it is to normalize the pathological levels of ADMA to abrogate its detrimental effects on NO bioavailability in the microvasculature and the bioenergetics of the target organ cells. Based on the studies on NO generated by iNOS in rodents, one may hypothesize that ADMA lowering could exacerbate hypotension. However, significant experimental evidence exists supporting that the risk of therapeutic enhancement of NO is unlikely. First, as previously discussed in the section on “NO Changes in Human Sepsis”, the high level of NO production in rodents is not consistent with the low NO levels observed in large animal species and humans. Second, the therapeutic enhancement of NO has been found safe in a variety of studies. For example, enhancing NO by overexpression of eNOS in transgenic animals has not produced detrimental effects. Rather, eNOS derived NO was protective in sepsis [71, 72]. Similarly, direct administration of sustained release NO-donor improved microvascular flow, capillary transit and 72-h survival of mice after LPS-induced endotoxemia [64]. Likewise, in salmonella typhimurium induced sepsis in iNOS-deficient mice showed aggravated hepatic and cardiovascular dysfunction and increased the risk of mortality as compared to the wild-type mice. Interestingly, pretreatment with a NO donor significantly attenuated these sepsis-associated abnormalities and improved survival, supporting that NO delivery offered protection [73]. A recent study in a severe hemorrhagic shock animal model showed that the efficacy of 7.5% saline and ALM (adenosine, lidocaine, magnesium) for resuscitation was blocked by NOS inhibitors, which produced a rapid fall in mean arterial pressure (MAP), cardiovascular collapse, sustained ventricular arrhythmias and 100% mortality. The study concluded that the ability of 7.5% NaCl ALM to resuscitate appears to be linked to NO-enhancing pathway [74]. In human studies, the administration of NO by inhalation in patients with sepsis has been found safe [63]. Similarly, inhaled delivery of NO in sever malaria patients was found safe [75]. More recently, NO has been safely used in several studies with COVID-19 patients [76].

Thus, a therapeutically controlled increase in NO such as by reducing ADMA to restore microvascular homeostasis does not appear to increase the risk of hypotension. Moreover, it is important to note that multiple mediators could contribute to the altered vascular hemodynamics in sepsis including NO, prostaglandins, endothelin, angiotensin II, platelet activating factor, thromboxane A_2_ and catecholamines. In addition, increased production of kynurenine, a product of tryptophan catabolism, has been shown to act as a systemic vasodilator and thus may contribute reduced diastolic blood pressure [77, 78]. Thus, based on the preponderance of the evidence for the detrimental consequence of NO inhibition and a lack of evidence for the adverse effect of restoring NO, the lowering of pathologic ADMA may be an efficacious and safe therapeutic approach.

## Conclusions

The evidence provided in this new perspective on role of host response-induced ADMA, which can contribute to the pathology of microvasculature as well as the target organs, suggests that the role of NO pathway in the progressive organ damage during sepsis needs re-examination. The recent preclinical demonstration that specific lowering of ADMA by pharmacological treatment is feasible and results in improved endothelial function, and reduction of inflammatory cytokine release, mitochondrial dysfunction and organ damage. Since persistent high ADMA levels exist in sepsis patients for a protracted period, its lowering may benefit during the progression phase of the disease where organ damage persists beyond the infectious phase. These findings support the investigation of a potential new therapeutic approaches to reduce progressive organ damage and mortality in sepsis.

## Data Availability

Not applicable.

## Abbreviations

ALM: Adenosine, lidocaine, magnesium
ADMA: Asymmetric dimethylarginine
DDAH: Dimethylarginine dimethylaminohydrolase
eNOS: Endothelial nitric oxide synthase
iNOS: Inducible nitric oxide synthase
mPTPs: Mitochondrial permeability transition pores
NO: Nitric oxide
NOS: Nitric oxide synthase
PAH: Pulmonary arterial hypertension
PHP: Pyridoxylated hemoglobin polyoxyethylene
ROS: Reactive oxygen species
TED: Therapeutic extracorporeal device
